# Alcohol-Related Behaviour in Freshmen University Students in Sardinia, Italy

**DOI:** 10.3390/ijerph18137203

**Published:** 2021-07-05

**Authors:** Alessandra Mereu, Arianna Liori, Claudio Dessì, Mariano Girau, Derrick Clifford Mc Gilliard, Alessandra Sotgiu, Roberta Agabio, Paolo Contu, Claudia Sardu

**Affiliations:** 1 Department of Medical Science and Public Health, University of Cagliari, 09042 Monserrato, Italy; amereu@unica.it (A.M.); dessiclaudio82@gmail.com (C.D.); mariano.girau1987@gmail.com (M.G.); derrick.mcg@gmail.com (D.C.M.G.); asotgiu@gmail.com (A.S.); contumail@gmail.com (P.C.); csardu@unica.it (C.S.); 2 Department of Biomedical Science, University of Cagliari, 09042 Monserrato, Italy; agabio@unica.it

**Keywords:** university students, alcohol, at-risk drinking, health promotion

## Abstract

This study aims to provide a picture of University of Cagliari students’ alcohol-related behaviour and to explore factors associated with it. Data were collected by administering a questionnaire to 992 freshmen university students from different programs consisting of twelve closed questions, including three questions from the Alcohol Use Disorders Identification Test for Consumption (AUDIT-C short form). Three subgroups of alcohol-related behaviour were distinguished (risky drinkers, social drinkers and abstainers). In order to explore factors associated with patterns of alcohol consumption, a multivariate logistic regression was performed. The prevalence of risky drinkers was 35%. A binge-drinking behaviour at least once in the last twelve months was declared by 65% (more widespread in men and in students living away from their parents). Risky consumption is significantly associated with age of onset of alcohol use, living away from parents’ home, drinking outside meals and attending health courses. Regarding the levels of daily alcohol consumption perceived as a health risk, 66% of men and 88% of women indicate values higher than those recommended. The results underline the need for tailored prevention measures. University could be a promising setting to implement actions according to a health promotion perspective, to empower students to control their alcohol consumption.

## 1. Introduction

Alcohol misuse is a serious concern in public health due to its effects on health, as this behaviour was the seventh-leading risk factor for premature death and disability globally in 2016 [1]. According to the “Global Status Report” on alcohol and health 2018 (World Health Organisation), in 2016, alcohol was responsible for 5.5% of all deaths and 5.1% of all DALYs (disability-adjusted life years) worldwide and caused 7.2% of all premature mortality (under the age of 69 years) in the same year [2]. The countries of the WHO European Region show the highest levels of alcohol consumption per capita worldwide [2,3]. Alcohol consumption among young adults is a relevant health problem because of its negative consequences; in the WHO European Region, 23.3% of all deaths in the 20–24-year-old age group were caused by alcohol [3].

Although Italy is one of the European countries with the lowest alcohol consumption, risky consumption is not an unknown phenomenon, affecting approximately 33% of young adults aged between 18 and 24 years [3,4]; this prevalence is higher in socially advantaged individuals with higher levels of education or no financial difficulties and in men, although the gender difference is narrowing due to increasing trends in women [4,5]. Furthermore, approximately 22% of men and 11% of women in the 18–24-year-old age group consumed 6 or more alcoholic units on a single occasion at least once in the last twelve months [5]. Several studies that involve university students showed the presence of a gender gap [6,7,8,9,10,11,12]. 

The risky use of alcohol is one of the leading causes of concern in the university student population [6,7,8,9,13,14,15,16,17,18]. Several studies have shown that alcohol misuse in university students is often related to increased levels of smoking [7,15,19], illicit drug use [7,15], physical harm [10,15], risky sexual behaviour [10,15,20,21], road traffic crashes [15,22,23] and poor academic performance [15,24,25,26]. The results of a recent study on Italian university students revealed that a relatively high proportion of respondents drove under the influence of alcohol and drugs, and 5% of them admitted to frequently driving drunk [9]. According to previous studies, factors like an early age of onset of consumption, before the age of 14 years [27], and drinking between meals [28,29] were associated with an increase of risky consumption.

The university years are a unique period in young people’s lives, they often leave their parents’ home, start managing their life and become independent, and it can be a stressful time in which the risk of engaging in dangerous behaviours, such as alcohol misuse, increases [30]. Literature has shown that, after beginning university, students increase consumption [17,31,32,33]; students who lived on their own, away from their parents’ control and support, showed higher prevalence of heavy alcohol consumption [7,14,34,35]. Previous studies on healthcare-profession students showed risky alcohol drinking behaviour [36,37,38,39,40]. Detecting alcohol consumption in this phase of life is crucial to plan prevention strategies tailored for university settings and to lead young adults towards drinking awareness.

Research conducted in Sardinia (Italy) at the University of Cagliari in 1997 suggested that alcohol consumption was a widespread habit among students, especially among men [13]. In Italy, Sardinia is one a region that deserves special attention; recent surveys, including all ages, show that, in Sardinia, the rates of risky and binge drinkers among men exceed Italian averages [5]. These data suggest that more in-depth analysis should be carried out in the subgroups potentially most exposed to this risk, such as university students. The present paper focuses on Sardinian students who attended their first academic year at the University of Cagliari to provide a picture of their alcohol-related behaviours and to explore factors associated with it. 

## 2. Materials and Methods

### 2.1. Data Collection

Data collection was performed in 2016, in April and May, at the public state University of Cagliari (Italy), which is the largest university in Sardinia. After receiving the consent of the Deans of the Faculties and of the University Ethics Committee, researchers administered questionnaires to freshman students in different courses: engineering, medicine, biology, economics, law and humanistic/humanities. The sample included only university freshmen in order to minimise the possibility of recall bias regarding changes in drinking behaviour following the start of university. The questionnaires, in agreement with tutors and lectures, were administered by researchers in classrooms before the beginning of lectures; students were not notified in advance of the survey to minimise the risk of potential information bias. In order to control ethical issues, the questionnaire was anonymous, and no sensitive data were collected; lecturers and tutors were not involved in the questionnaire’s administration or subsequent collection, to reassure students regarding their total anonymity. Researchers specified that participation was voluntary and that those who did not wish to participate could return the questionnaire not completed. The proportion of students who did not agree to participate in the survey and returned a questionnaire without completing it was <5%. Questionnaires completed by students older than 25 were excluded from the data analysis. 

### 2.2. Questionnaire

Students’ alcohol behaviour was assessed through a questionnaire that included twelve closed questions. After questions related to age and gender, the questionnaire included the three questions from the Alcohol Use Disorders Identification Test for Consumption (AUDIT-C short form) to identify people with risky consumption [41,42,43]. The definition of alcoholic drink, an “alcoholic drink contains 12 g of alcohol which is found in 330 mL of beer or 150 mL of wine or 40 mL of spirits”, was indicated on the title page of the questionnaire. Additional questions explored factors potentially related to students’ drinking habits:

- Living with parents (yes or no).
- Attending biological/health classes (yes or no).
- Age of the first consumption of an alcoholic drink (continuous variable).
- Changes in alcohol consumption after beginning university (one answer to be chosen from “no change”, “increase” and “decrease”).
- Drinking alcohol outside meals (yes versus no).
- Reasons for drinking alcohol: the good taste of alcoholic beverage (yes versus no), moments of conviviality, to have a good time/for fun (yes versus no), to relax (yes versus no), to socialise (yes versus no), to feel confident (yes versus no), in moments of difficulty/stress (yes versus no).
- Negative experienced alcohol-related consequences: general malaise (yes/no), doing something you regret (yes versus no), car crashes (yes versus no), penalties for driving under the influence of alcohol (yes versus no), need for medical assistance (yes versus no), involvement in violent incidents (yes versus no).
- The level of daily alcohol consumption perceived as a health risk (one answer to be chosen from 1, 2, 3, 4 or more alcoholic units).

### 2.3. Data Analysis

The data from the AUDIT-C questions and from the last question on the level of daily alcohol consumption perceived as a health risk were interpreted in agreement with the guidelines of the Italian Health Institute. In the adult population, 2 alcohol units a day for men and 1 alcohol unit a day for women are the consumption risk thresholds. The cut-off value of AUDIT score ≥5 indicates risky consumption for men, while an AUDIT score ≥4 indicates risky consumption for women. The score of the AUDIT-C scale allows three subgroups of alcohol-related behaviour to be distinguished: the risky consumption or risky drinkers, the low-risk consumption or low-risk drinkers (hereinafter referred to as social consumption/social drinkers) and the abstainers, who declared that they never consumed alcohol. Binge drinking was defined as the consumption of 6 or more alcoholic units on a single occasion at least once in the last twelve months [5]. 

Students’ alcohol behaviour and related factors were described through percentages with 95% confidence intervals (C.I. of 95%) in the case of qualitative variables or with means and standard deviations (SD) in case of quantitative variables. 

In order to explore factors associated with patterns of alcohol consumption and assess potential interactions/confounding effects, a multivariate logistic regression was carried out. The dependent variable has two categories, risky consumption versus social consumption. Gender, age of onset of alcohol use, living away from parents’ home (yes versus no), drinking outside meals (yes versus no) and attending biological health university courses (yes versus no) were considered independent variables. The final regression model included only significant associated variables. 

The frequencies of different reasons for drinking alcohol, as well as the frequencies of reported negative consequences after drinking, were compared between risky and social drinkers through chi-square tests. Analogously, the chi-square test was used to explore the association between gender and the level of daily alcohol consumption perceived as a health risk.

Statistical analysis was performed using SPSS software (version 20; SPSS Inc., Chicago, IL, USA).

## 3. Results

The final sample consisted of 992 people (18–25 years old) with a mean age of 20.9 years (SD 1.4); 42% were men (C.I 39–45%); 43% (C.I. 40–46%) of students lived away from parents, and 38% (C.I. 34–41%) of students attended health courses. According to the results of the AUDIT-C short-version scale, the prevalence of risky drinkers among university students in Cagliari was 35% (C.I. 32–38%); the prevalence of social drinkers was 58% (C.I. 55–61%), and the prevalence of abstainers was 7% (C.I. 5–9%). Binge-drinking behaviour at least once in the last twelve months was declared by 65% of the total sample (C.I. 62–68%). Binge-drinking attitudes were significantly more widespread in men (76%, C.I. 72–80%) than in women (57%, C.I. 53–61%) and in students living away from their parents (72%, C.I. 68–76%) than in those living with their parents (59%, C.I. 55–63%). 

The mean age of the first consumption of an alcoholic drink was 15 years (SD 1.9). A total of 17% (C.I. 14–19%) of students stated that they had increased alcohol consumption after beginning university. In particular, the increase in alcohol consumption was significantly more common in students who lived away from their parents’ home (23%, C.I. 19–27%) than in those who lived with their parents (12%, C.I. 9–15%). 

The results of multivariate regression exploring factors associated with drinking habits revealed no significant interaction effects. Gender was not significantly associated with risky consumption, and it was not a confounder; therefore, it was deleted from the model. The final regression models included four variables significantly associated with drinking behaviour (Table 1). The probability of risky consumption decreased with increasing age of the first intake of a glass of alcoholic drink (OR 0.70). Additionally, students who lived away from their parents’ home had a 2.34 times greater likelihood of risky consumption. Drinking outside meals increased the probability of risky consumption 3.74 times. Students attending biological/health courses had greater likelihood of being risky drinkers than students attending other courses (OR 1.66).

Table 2 describes the results of the question exploring drinking reasons among social and risky drinkers; for this question, giving more than one answer was allowed. The differences between social and risky drinkers were significant for almost all the explored items: because of the good taste of alcoholic beverages (66% for risky drinkers, 50% for social drinkers), during social moments for the pleasure of conviviality (70% for risky drinkers, 61% for social drinkers), out of the desire to have a good time (47% for risky drinkers, 21% for social drinkers), to relax (22% for risky drinkers, 9% for social drinkers), with the aim of socialising (16% for risky drinkers, 8% for social drinkers) and in stressful moments (11% for risky drinkers, 3% for social drinkers). The only item that did not show a statistically significant difference between the two groups was “to feel confident” (14% for risky drinkers, 12% for social drinkers).

Table 3 describes reported negative alcohol-related consequences among social and risky drinkers; giving more than one answer to this question was allowed. The results highlight a significant difference between social and risky drinkers for several consequences, such as general malaise (64% for risky drinkers, 35% for social drinkers), doing something they regretted (21% for risky drinkers, 8% for social drinkers) and involvement in violent episodes (7% for risky drinkers, 1% for social drinkers).

Table 4 summarises the results of the question regarding the levels of daily alcohol consumption perceived as a health risk according to gender; 66% of men and 88% of women indicate values higher than recommended and show that they are not aware of the real risk threshold for their own gender (2 alcohol units a day for men and 1 alcohol unit a day for women in the adult population). The lack of awareness around this topic is an issue that is significantly more prevalent in women students.

## 4. Discussion

At-risk drinking is a major cause for concern [6,15,16,17,28,36,44,45]. From recent research on patterns of alcohol consumption in European university students, it emerged that Italian participants showed low-risk drinking behaviour compared to Nordic European students [18]. The different methodologies of alcohol consumption assessment and the limited number of studies in the Italian university context make it difficult to compare results [8,9,41]. However, the prevalence rate of risk consumption among university students that emerged in this study (35.3%) highlights the relevance of this topic. The problem of alcohol use among students at Cagliari University, which has been studied since 1997, is still relevant even after 20 years, emphasising the urgent need for tailored prevention measures [13].

It is common knowledge that there is a gender gap in alcohol consumption; fewer women are current drinkers, and they drink less than men in terms of volume, although the number of current-drinker women is increasing worldwide [2,46]. Many studies conducted among university students have confirmed the presence of this gender gap [6,7,8,9,10,11,12]. In Europe, this gender gap is greater in Mediterranean and Eastern European countries; regarding current-drinker prevalence, the gap was consistently high across all age groups, and the narrowest gender gap was found in young adults (25–34-year-olds) [3]. Our results show that the indicator “risky consumption”, calculated according to AUDIT-C with different cut-offs for men and women, does not appear to be influenced by gender. This outcome is in line with some recent studies, mostly from Nordic European countries, that showed a decrease in the difference between genders regarding alcohol consumption [15,16,34,45,47]. Instead, as far as the indicator “binge drinking” is concerned, our results bring to light a gender gap, with this behaviour significantly more widespread in men than in women. However, we cannot overlook that our assessment of binge drinking through the third question of AUDIT-C was based on the same cut-off for both sexes (6 or more alcoholic units on a single occasion). Consequently, the lower prevalence of binge-drinking behaviour detected in women does not necessarily correspond to a lower risk for this subgroup, since women are more susceptible to the effects of alcohol [48,49].

In our research, risky consumption was significantly associated with age of onset of alcohol use, and the mean age of the first consumption of an alcoholic beverage was 15 years old; this finding is similar to previous studies [9,10,50]. An early age of onset of consumption of alcoholic beverages, before the age of 14-years-old, was associated with an increased risk for alcohol dependence and abuse at later ages; thus, it was a predictor of possible impaired health status [27].

In line with previous studies, our research showed that 17% of students declared that they have increased consumption since beginning university [17,31,32,33]. During the initial period at university, in which young adults begin managing their lives outside of their family context, the risk of engaging in hazardous behaviours acquired in teenage years or of having new experiences, such as misuse of alcohol, can increase [9,34,51,52]. In particular, the freshman year is a period in which young students are focused on integrating into a new environment and building new connections and friendships, and this process can frequently involve drinking [17]. Furthermore, in the present study, a greater risk of risky consumption and binge drinking emerged among those students who lived away from their parents’ home; this result is in line with previous studies that found higher rates of heavy alcohol consumption in students who lived on their own and showed that living in a situation away from parental control and support increased the risk of consuming alcohol more frequently [7,14,34,35]. A higher level of parental support seemed to be negatively associated with alcohol abuse and was a protective factor for alcohol-related harms [34,50]. Drinking between meals was also a factor that increased risky consumption (O.R. 3.74), as shown in previous studies [28,29]. Our research highlights that a high proportion of risky drinking occurred among students who attended healthcare-profession courses (O.R. 1.62). Multiple studies on healthcare-profession students [36,37,38,39,40] have found evidence of problematic alcohol drinking behaviour, an issue that, in some studies, have shown increases over the years [12,53,54]. The difficult adjustment to this kind of study, with a major stress burden and a large workload, can be one of the reasons for these findings [55].

Concerning the main reasons for drinking, in our sample, as in other studies, students stated that they drink at social gatherings, for the pleasure of conviviality, with the intent to have a good time and because they like the taste of alcoholic beverages [7,10,28]. Alcohol consumption is an integral part of student’s lives and a common practice to integrate into a new environment, with the purpose of creating social bonds, making new friends and having fun [17]. In Italian culture, drinking alcoholic beverages is a social habit; alcohol consumption is intrinsic to Italian social custom and has relative social acceptation, mostly during daily meals and special family gatherings. The connection between the reproduction of existing alcohol consumption habits in young adult generations and a country’s cultural heritage has been highlighted in several studies [17,56]. A source of concern is that this connection between community life and alcohol consumption might lead to the social exclusion of those students who abstain for multiple reasons, such as religious, cultural or health-related factors [17]. Related to the question about negative alcohol-related consequences, in line with previous studies, among risky drinkers, the majority of repercussions were general malaise, with minor proportions regretting something they have done [10,15] and experiencing involvement in violent episodes [10].

It is important to highlight that, in the present study, a large proportion of respondents show a misguided perception of the alcohol consumption risk thresholds for their own gender. This aspect seems to be more relevant in women, for whom the threshold is lower than for men. This finding is particularly significant because it reveals a relevant lack of knowledge about alcohol consumption risk thresholds that can lead easily and unknowingly to alcohol-related risky behaviours. Similar results were observed in a recent Italian study that highlighted how young adults were confused about the concept of moderate alcohol consumption and the contents of alcohol per drink and were not informed on the legal alcohol limit to drive after drinking [28]. This lack of awareness results in even greater challenges in light of a recently published systematic analysis that emphasised how safe alcohol limits need to be revaluated; the safest level of alcohol consumption is none, since the protective effects on ischaemic heart disease and diabetes suggested by some past studies were offset when total alcohol-attributable health risk was considered [1]. Surely this finding deserves particular attention and may be the subject of further studies.

The present study offers a detailed picture of alcohol-related behaviour in freshmen university students; however, some limits should be recognised. The sample included only university freshmen students from the University of Cagliari; therefore, the results may be not fully representative of students attending subsequent years or of students from other universities. Additionally, we cannot be sure that all respondents answered truthfully; however, the method of data collection, which guaranteed anonymity, minimised the risk of this bias.

## 5. Conclusions

University students deserve special public-health attention and tailored strategies that favour healthy alcohol-related behaviour. Students living away from their parents and students attending health courses are the most vulnerable subgroups, while women currently appear as vulnerable as men. A relevant lack of awareness about alcohol risk thresholds emerged from this research, particularly in women. Risky drinkers in the university context represent a worthy target for prevention to decrease the risk of negative consequences of alcohol misuse. The university is a promising setting to implement global actions with a health-promotion perspective to empower students to control all aspects of their lives, including alcohol consumption. 

## Figures and Tables

**Table 1:** Multiple logistic regression analysis: factors associated with drinking habits (risky consumption versus low risk consumption).

	*p*	O.R.	I.C. 95% OR
Age of onset of alcohol use	<0.0001	0.70	0.64–0.77
Living away from home versus living with family	<0.0001	2.34	1.72–3.19
Drinking outside meals versus not drinking outside meals	<0.0001	3.74	2.74–5.12
Attending biological/health courses versus others	<0.002	1.66	1.22–2.28

**Table 2:** Frequencies of reported drinking reasons in social and risk drinkers.

Drinking Reasons	Social Drinkers	Risky Drinkers	Total Sample	*p* Value
The good taste of alcoholic beverage	50%	66%	55%	<0.001
Moments of conviviality	61%	70%	62%	<0.005
To have a good time/for fun	21%	47%	30%	<0.001
To relax	9%	22%	13%	<0.001
To socialise	8%	16%	10%	<0.001
To feel confident	12%	14%	12%	0.375
In moments of difficulty/stress	3%	11%	6%	<0.005

**Table 3:** Frequencies of reported negative alcohol-related consequences in social and risky drinkers.

Negative Alcohol-Related Consequences	Social Drinkers	Risky Drinkers	Total	*p* Value
Nothing	55%	25%	45%	<0.001
General malaise/discomfort	35%	64%	45%	<0.001
Doing something they regretted	8%	21%	13%	<0.001
Car crashes	1%	1%	1%	0.29
Penalties for driving under the influence of alcohol	1%	2%	1%	0.03
Health care treatment required	1%	3%	1%	0.008
Involvement in violent episodes	1%	7%	3%	<0.001

**Table 4:** Percentages of students aware of the risk threshold of alcohol consumption for their gender.

	Men	Women	*p* Value
Students aware of the risk threshold for their gender	34%	12%	<0.001
Students unaware of the risk threshold for their gender	66%	88%	

## Data Availability

The dataset used during the current study is available on reasonable request from the corresponding author.
